# Broadband Tamm Plasmons in Chirped Photonic Crystals for Light-Induced Water Splitting

**DOI:** 10.3390/nano12060928

**Published:** 2022-03-11

**Authors:** Maxim V. Pyatnov, Rashid G. Bikbaev, Ivan V. Timofeev, Ilya I. Ryzhkov, Stepan Ya. Vetrov, Vasily F. Shabanov

**Affiliations:** 1 Kirensky Institute of Physics, Krasnoyarsk Scientific Center, Siberian Branch, Russian Academy of Sciences, 660036 Krasnoyarsk, Russia; bikbaev@iph.krasn.ru (R.G.B.); tiv@iph.krasn.ru (I.V.T.); s.vetrov@inbox.ru (S.Y.V.); shabanov@ksc.krasn.ru (V.F.S.); 2 Siberian Federal University, 660041 Krasnoyarsk, Russia; rii@icm.krasn.ru; 3 Institute of Computational Modelling, Krasnoyarsk Scientific Center, Siberian Branch, Russian Academy of Sciences, 660036 Krasnoyarsk, Russia

**Keywords:** water splitting, plasmon catalysis, solar-to-hydrogen efficiency, photocurrent

## Abstract

An electrode of a light-induced cell for water splitting based on a broadband Tamm plasmon polariton localized at the interface between a thin TiN layer and a chirped photonic crystal has been developed. To facilitate the injection of hot electrons from the metal layer by decreasing the Schottky barrier, a thin n-Si film is embedded between the metal layer and multilayer mirror. The chipping of a multilayer mirror provides a large band gap and, as a result, leads to an increase in the integral absorption from 52 to 60 percent in the wavelength range from 700 to 1400 nm. It was shown that the photoresponsivity of the device is 32.1 mA/W, and solar to hydrogen efficiency is 3.95%.

## 1. Introduction

The current reduction of fossil fuel reserves generates a need for the development of alternative, primarily renewable, energy sources. The methods that use the solar radiation energy evoke special interest. Today, the investigations in the field of solar power are mainly focused on two areas: the creation of solar cells (batteries) for converting the sunlight energy directly into electricity, and the development of a direct converter of the solar energy into the energy of chemical carriers, such as hydrogen.

Hydrogen is an ideal element for transport, storage, and generation of electricity with zero carbon emissions, since, when pure hydrogen is used as a fuel, only the water vapor is generated. The energy of combustion of a kilogram of hydrogen corresponds to approximately 2.1 kg of natural gas or 2.7 kg of high-octane gasoline. The use of hydrogen in transportation fuel cells is almost 200% more efficient than the use of gasoline. The use of hydrogen in internal combustion engines increases the efficiency of the latter by almost 70% as compared with gasoline engines.

It is well known that, in nature, free hydrogen sources are lacking. Free hydrogen is obtained from hydrogen-containing compounds. In this case, the expediency of using hydrogen as a fuel source can only be justified if the energy consumption for hydrogen production is lower than the energy released during its use. Therefore, the use of hydrogen as a fuel is complicated. The possibility of the direct photodegradation of water into hydrogen and oxygen by solar energy is a promising solution to this problem.

The operation of the photoelectrochemical devices used for water splitting is based on the electron-hole separation with the additional oxygen or hydrogen evolution half-reactions. The reduction and oxidation half-reactions occur on the cathode and anode surfaces, respectively, in an aqueous electrolyte, which closes the current circuit between the electrodes [1,2,3]. The idea is analogous to that underlying the electric current generation under the action of solar radiation [4,5]. The fundamental difference is that the conversion of the electrical energy into the useful work can, in principle, be performed at any voltage. To decompose water, it should theoretically be at least 1.23 eV, i.e., higher than the thermodynamic redox potential for overall water splitting. In reality, this value, with regard to the loss, is ∼2.3 eV. The overpotential losses caused due to the impedances and photocorrosion, and the kinetic and mass-transport losses caused due to the generation of gas bubbles at the electrode surfaces, raise a potential barrier for a successful water-splitting reaction [6,7].

The first priority in water splitting is fabrication of the structures with the high solar energy conversion efficiency. A series of photocatalytic designs for producing pure hydrogen have been proposed, in which different solar radiation ranges are used. A promising way of increasing the efficiency of light-induced water splitting is the introduction of plasmon-active nanostructures into the design of photocatalysts [8,9,10,11,12,13,14,15,16]. Such devices are developed to increase the oxidation photocurrent induced by the injection of hot electrons created by plasmons into a catalytic medium. This effect can be obtained by increasing the absorption of light incident onto a structure.

The absorption properties of the structures based on Tamm plasmon polaritons have been demonstrated many times [17,18,19,20,21]. The Tamm plasmon polariton (TPP) is a mode localized between the metal and Bragg mirrors, which manifests itself as a resonance line in the energy spectra of a structure. Recently, the concept of a broadband TPP localized at the interface between a Bragg reflector and a strongly absorbing metal (Al, Cr, or Ti) has been proposed [22]. In such a broadband TPP, the width of the resonance in the spectrum is comparable to the Bragg reflector band gap.

Recently, basing on this idea, a broadband infrared planar hot-electron photodetector has been proposed, which consists of a silicon substrate, a Bragg reflector, and two thin (8 nm) silicon and titanium nitride layers [23]. The solar radiation falls onto TiN. The comparison of the absorption properties of the Au- and TiN-based structures convincingly demonstrated the superiority of the latter as an absorption element.

In this study, we discuss an improved design of an analogous structure, in which a regular Bragg mirror with a constant period is replaced by a chirped mirror [24] with the period varying linearly with increasing distance from the TiN layer. The chirping is aimed at the broadening of the spectral region of the multilayer mirror reflection and, consequently, of the absorption region of the TPP. We expect that chirping will ensure the more efficient light harvesting, increase the photocurrent of water oxidation, and ultimately enhance the solar-to-hydrogen efficiency.

## 2. Design of the Structure

**Figure 1:**
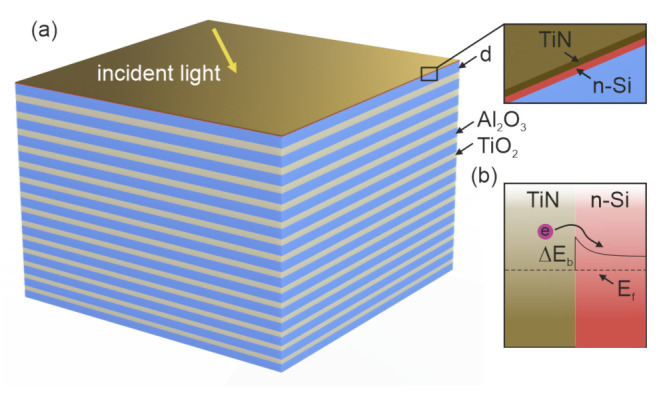
(**a**) Schematic of an electrode for light-induced water splitting: top TiN layer, semiconductor, and chirped PhC. (**b**) Energy band diagram of the TiN/Si structure with barrier height $\Delta E_{b}$ .

The calculation was made using the Berreman 4×4 matrix method [25]. For the light propagating along the *z*-axis with frequency ω , the equation is ∂ψ/∂z=(−iω/c)Δψ , where $\psi \left(z\right)={\left(E_{x},H_{y},E_{y},-H_{x}\right)}^{T}$ and Δ(z) is the Berreman matrix, which depends on the permittivity and incident wave vector. We numerically simulated the optical properties of a planar structure (Figure 1a), which can be used as one of the electrodes of a photoelectrochemical cell. Its components, from top to bottom, are the TiN thin film with a thickness of $d_{m}=15$ nm, a thin semiconductor (n-Si) layer with a thickness of 8 nm, and a photonic crystal (PhC). The thickness of the n-Si layer was taken according to the phase matching condition, which provides excitation of the Tamm plasmon polariton in the center of the PhC band gap. The design and materials of the elements used are as in Wang’s study [23]. We assumed that the structure is surrounded by a medium with a refractive index of 1.33. In contrast to [23], in our design the PhC was formed by 15 pairs of the TiO ${}_{2}$ ( n=2.4 ) and Al ${}_{2}$ O ${}_{3}$ ( n=1.7 ) layers. The number of layers was increased to ensure a smoother change in the period upon chirping. An additional Al ${}_{2}$ O ${}_{3}$ layer with a thickness of d=120 nm is located between the silicon layer and the first TiO ${}_{2}$ layer. The optical constants of TiN and n-Si were taken from the Palik’s database [26]. Thus, the dispersion of the refractive index for the TiO ${}_{2}$ and Al ${}_{2}$ O ${}_{3}$ layers was disregarded, as in [23]. The layer thicknesses differ from the values presented in the original work, because we optimized the parameters with respect to an increase in the integral absorbance, which is discussed below.

**Figure 2:**
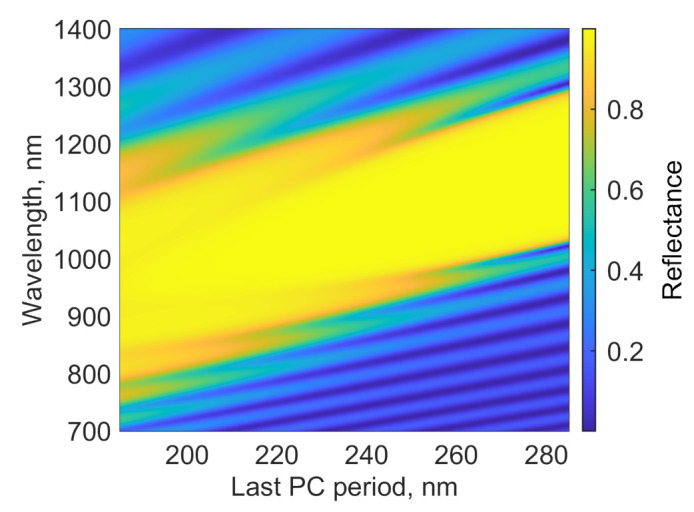
Reflection of the chirped photonic crystal at different final periods; the initial period is 285 nm (the thicknesses of the TiO ${}_{2}$ and Al ${}_{2}$ O ${}_{3}$ layers are 115 and 170 nm, respectively) and d=120 nm.

The doped n-type Si makes it possible to obtain a low value of the Schottky barrier at the boundary with titanium nitride, which facilitates the injection of hot electrons from the metal layer. A Schottky barrier in this case is $\Delta E_{b}=0.35$ eV [27]. The energy diagram is presented in Figure 1b. PhC chirping is a linear change in its period with the depth of the structure. As a result of chirping, the Bragg frequency gradually changes along the PhC, which leads to the expansion of the reflection spectral region. Figure 2 shows the reflection spectrum of a bare PhC with a period that decreases with increasing distance to the radiation source. The initial period, as in [23], is 285 nm, and the thicknesses of the initial TiO ${}_{2}$ and Al ${}_{2}$ O ${}_{3}$ layers are 115 and 170 nm, respectively. The final period is 185 nm, and the thicknesses of the final TiO ${}_{2}$ and Al ${}_{2}$ O ${}_{3}$ layers are 65 and 120 nm, respectively. It can be seen that the chirping shifts and broadens the photonic band gap of the reflector. In addition, the chirping increases the bandwidth of Tamm plasmon conjugated with the metal layer. This should enhance the absorption in the investigated model and the efficiency of hot-electron generation for the photocatalytic applications.

The structure from Figure 1a containing the PhC with a period decreasing from 285 nm (the thicknesses of the TiO ${}_{2}$ and Al ${}_{2}$ O ${}_{3}$ layers are 115 and 170 nm, respectively) to 195 nm (the thicknesses of the TiO ${}_{2}$ and Al ${}_{2}$ O ${}_{3}$ layers are 75 and 120 nm, respectively) with increasing distance from the radiation source is hereinafter referred to as a chirped structure, and the structure containing the PhC with a period of 285 nm is referred to as a regular structure.

**Figure 3:**
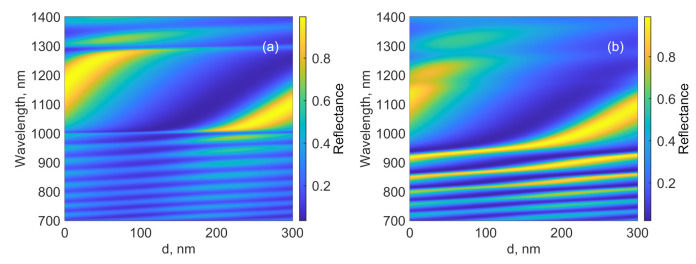
Reflection of (**a**) the regular and (**b**) chirped structures at different *d*.

The conjugation of the PhC with the thin Si and TiN layers leads to the formation of a broadband Tamm plasmon [22] at the metal–semiconductor interface. The incident light is confined between the mirror and the top of the structure. A characteristic dip is formed in the reflection spectrum of the structure. The position of the dip significantly depends on the thickness of the Si layer and the first layer of Al ${}_{2}$ O ${}_{3}$ with thickness *d* (see Figure 1a). Then we consider the effect of thickness *d*. Figure 3 shows the reflection spectra of the structures with the regular and chirped mirrors at different *d* values. The comparison of the spectra shows that, in the case of the chirped structure, the dip is wider than in the structure with a regular mirror. In addition, peaks corresponding to the PhC edge modes appear. The reflection at the frequencies of the edge modes is significant; in particular, at the frequency of the first short-wavelength edge mode, it attains 98% (945 nm at d=120 nm). The simulation of the inverse structure with a period increasing with the distance from the light source showed that, in this case, the edge modes change their position in the spectrum for a longer-wavelength one with respect to the Bragg frequency.

The layered structure can be fabricated by various layer formation methods, e.g., atomic layer deposition, magnetron sputtering, or low-pressure chemical vapor deposition. The TiO ${}_{2}$ and Al ${}_{2}$ O ${}_{3}$ layers obtained in this way will be polycrystalline, so, in this study, the material anisotropy is ignored.

## 3. Absorption Properties of the Structure

An important characteristic in the estimation of the absorption properties is the integral absorption, which is the absorption in the absorbing layer normalized to the solar radiation spectrum [28,29,30].

(1) $$A_{total}=\frac{\int_{\lambda_{1}}^{\lambda_{2}}A\left(\lambda \right)S\left(\lambda \right)d\lambda }{\int_{\lambda_{1}}^{\lambda_{2}}S\left(\lambda \right)d\lambda },$$

where $\lambda_{1}$ and $\lambda_{2}$ are the boundaries of the spectral region, A(λ) is the absorption of the structure, which can be determined through the transmission and reflection coefficients of the structure [31,32], and S(λ) is the solar radiation spectra (AM1.5). In the structure under study, the incident light trapped between the TiN/semiconductor interface is mainly absorbed by the metallic film and facilitates the hot-electron generation. This is due to the fact that, in the investigated spectral region, the imaginary part of the refractive index of the Bragg reflector layers is indistinguishable from zero.

**Figure 4:**
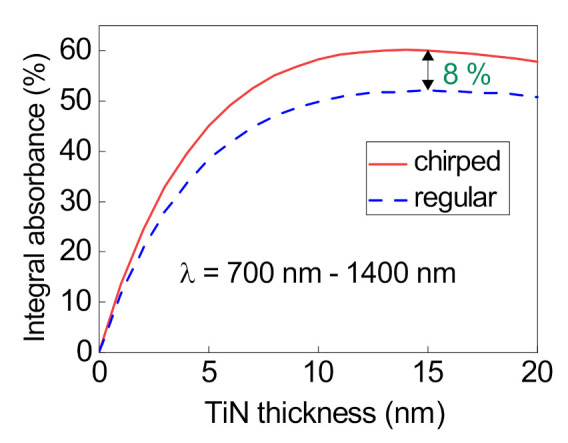
Integral absorbance in the regular and chirped structures in the range from 700 to 1400 nm at different TiN layer thicknesses $d_{m}$ .

In Figure 4, the absorbances in the regular and chirped structures at different TiN layer thicknesses $d_{m}$ are compared. In Wang’s study [23] the external medium is air; in contrast, we consider the case of an external medium refractive index of 1.33 and more reflector periods—therefore, the maximum integral absorbance in both cases is observed at $d_{m}=15$ nm. At the normal incidence of light at such a thickness $d_{m}$ , the integral absorption in the range from $\lambda_{1}=700$ nm to $\lambda_{2}=1400$ nm increases from 52% for the regular reflector to 60% for the chirped one.

**Figure 5:**
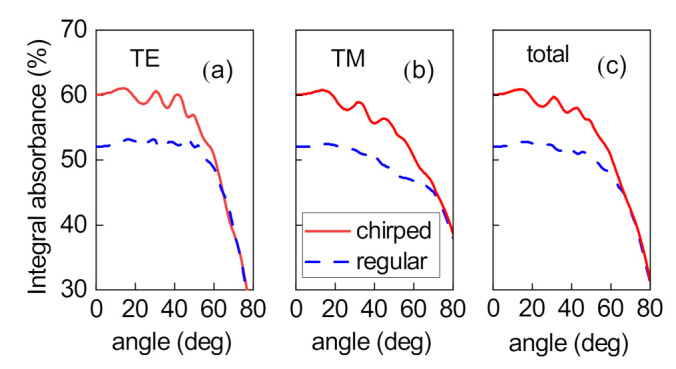
Integral absorbance of the regular and chirped structures at different angles of light incidence for (**a**) the TE and (**b**) TM polarization of light and (**c**) total integral absorbance.

The photoelectrochemical cell should provide a high integral absorbance index over the entire angular range of the incident light emission. In the general case, the integral absorbance is determined for each of the linear polarizations of the incident light separately and their arithmetic mean $A_{total}=\left(A_{TE}+A_{TM}\right)/2$ yields the total absorbance on the layer. Under the normal incidence, we have $A_{total}=A_{TE}=A_{TM}$ . We made the comparative calculation of the integral absorbance of the structure for the TE (Figure 5a) and TM (Figure 5b) linear polarizations of the incident light, as well as the total integral absorbance (Figure 5c). The advantage of the chirped structure is valid at arbitrary angle of light incidence.

Hot electrons generated in the metal move due to the absorption of photons. The electron energy exceeds the Fermi level of the metal, and the electrons enter the conduction band of the neighboring semiconductor. Only the hot electrons crossing the barrier formed at the metal–semiconductor interface can induce a photocurrent. The hot-electron generation is affected by the absorption of light by the metals. The high absorption in a wide range effectively increases the photocurrent sensitivity. In the proposed planar structure, it is assumed that the momentum distribution of hot electrons in the metal layer is isotropic. We assume that the semiconductor collects the electrons generated inside the TiN without annihilation or release in air. Thus, the probability of electron transport is unity. It should be noted that, in the proposed structure, the TiN layer is fairly thin; therefore, the thermalization of electrons and electron–phonon scattering are not taken into account. On the contrary, when the absorbing metal layer is thick, the thermalization of hot electrons and the effect of electron-phonon scattering should be taken into account [33,34].

The occurrence of the photocurrent in the investigated model can be understood by considering the hot electron transport through the Schottky barrier using a three-stage model [35,36]:

1. Plasmons nonradiatively decay into hot electrons;
2. Hot electrons are transferred to the metal–semiconductor interface prior to thermalization;
3. Hot electrons are injected into the conduction band of the semiconductor through internal photoemission.

To calculate the photoresponsivity, we used the Fowler model [37]. The photoresponsivity is a function of absorption in a metal [38]:

(2) R(ω)=q×A(ω)×η/ℏω,

where *q* is the elementary charge, ℏω is the photon energy, and A(ω) is the absorbance coefficient. The internal quantum yield η indicates the possibility of injection of hot electrons and is determined as

(3) $$\eta =\frac{{\left(\hbar \omega -\Delta E_{b}\right)}^{2}}{4E_{f}\times \hbar \omega }.$$

**Figure 6:**
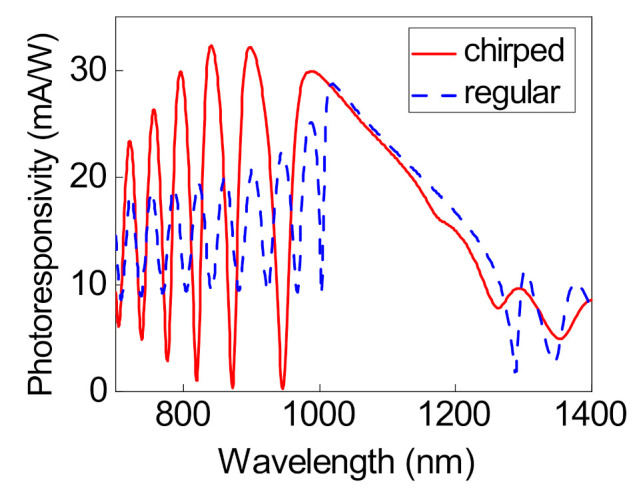
Comparison of the photoresponsivity in the regular and chirped structures at different incident radiation wavelengths.

It depends on the Fermi level $E_{f}$ of the metal and the Schottky barrier $\Delta E_{b}$ between the metal and semiconductor. The Schottky barrier formed between TiN and n-Si is 0.35 eV and the Fermi level of TiN is 4.2 eV [19]. The distribution of the photoresponsivity is plotted at different wavelengths in Figure 6. The maximum value is 32.1 mA/W at wavelengths of 897 and 840 nm, which correspond to the first and second short-wavelength edge modes.

The obtained photoresponsivity was compared with the photoresponsivity of the other Tamm plasmon polariton based devices. The results are presented in Table 1. It can be seen from this table that the proposed structure provides the greatest photoresponsivity.

The overall solar-to-hydrogen (STH) efficiency is given by [32]:

(4) $$\eta_{STH}=R\left(V_{redox}-V_{bias}\right)\cdot 100\%$$

where $V_{redox}$ is usually taken to be 1.23 V (at room temperature), based on Gibbs free energy change for water splitting of 237 kJ/mol and $V_{bias}$ is the bias voltage. In the case of zero bias voltage, $\eta_{STH}=3.95\%$ . We believe that the obtained results are promising for design of photoelectrochemical devices.

## 4. Conclusions

In this research, we proposed a design of an electrode of the photoelectrochemical cell based on a chirped photonic crystal and a thin titanium nitride layer separated by a semiconductor layer. The Tamm plasmon can be excited in the structure, and the corresponding broadband resonance appears in the absorption spectrum of the structure. Replacing the regular Bragg reflector by the chirped mirror allowed us to broaden the photonic band gap and, consequently, the absorption range of the structure. In addition, chirping leads to an increase in the absorption at the frequencies corresponding to the edge modes of the structure. The use of the chirped reflector made it possible to increase the integral absorbance of the structure from 52% to 60% in the region from 700 to 1400 nm, and the photoresponsivity attained 32.1 mA/W at the frequencies of the first and second edge modes. We expect that the proposed structure will be used as an electrode of a photoelectrochemical cell for light-induced water splitting and obtaining pure hydrogen. In addition, the proposed structure can be applied in the other photon harvesting areas, including photodetection, photocatalysis, and photovoltaics. Note that the Tamm modes in the chirped structures were predicted here for the first time.

## Figures and Tables

**Table 1:** Photoresponsivity of different types of TPP-based devices.

Photoresponsivity (mA/W)	Source
2.35	Shao et al. [39]
15.9	Zhang et al. [40]
21.87	Liang et al. [41]
26.1	Wang et al. [23]
**32.1**	**proposed structure**

## Data Availability

The data presented in this study are available upon reasonable request from the corresponding author.
